# Evaluating pellet and mash rumen protected soybean groat on nutrient digestibility, animal performance, carcass characteristics, and meat quality of fat-tailed sheep

**DOI:** 10.14202/vetworld.2025.976-985

**Published:** 2025-04-25

**Authors:** Joko Riyanto, Ahmad Pramono, Susi Dwi Widyawati, Sudibya Sudibya, Muhammad Cahyadi, Windi Nur Yuliana, Alfian Andi Apriyanto, Gebby Rosita Jolanda Putri, Farouq Heidar Barido

**Affiliations:** 1 Department of Animal Science, Faculty of Animal Science, Universitas Sebelas Maret, Surakarta, Central Java, 57126, Indonesia; 2 Halal Research Center and Services, Institute for Research and Community Service, Universitas Sebelas Maret, Surakarta, Central Java, 57126, Indonesia

**Keywords:** carcass characteristics, fat-tailed sheep, feed form, meat quality, nutrient digestibility

## Abstract

**Background and Aim:**

Optimizing feed strategies is critical in livestock production to enhance animal performance, nutrient utilization, and meat quality. Feed form, such as pelleted, mash, or blended forms, significantly influences these parameters. Investigating the optimal feed form for fat-tailed sheep production can improve economic outcomes and meat quality. This study aimed to evaluate the impact of dietary feed forms – pelleted (P10), mash (M10), and a blended form consisting of 50% pellet and 50% mash (M5P5) – on nutrient digestibility, animal performance, carcass characteristics, and meat quality attributes in fat-tailed sheep.

**Materials and Methods:**

Fifteen fat-tailed lambs were randomly allocated to three experimental groups receiving either 100% mash feed (M10), a 50:50 mixture of mash and pelleted feed (M5P5), or 100% pelleted feed (P10) for a 90-day feeding trial. Feed intake parameters (dry matter, organic matter, crude protein, crude fat, crude fiber, and nitrogen-free extract) were recorded. Nutrient digestibility was assessed, and production performance measures, including body weight gain, average daily gain (ADG), feed efficiency ratio (FER), and feeding cost per gain were determined. Post-slaughter carcass traits, proximate meat composition, cholesterol content, pH, water-holding capacity (WHC), cooking loss, and shear force values were evaluated.

**Results:**

Dietary feed forms had no significant impact (p > 0.05) on feed intake, nutrient digestibility, ADG, or FER. However, significant differences (p < 0.05) emerged in carcass traits, notably with increased hot carcass weights observed in M5P5 (19.57 kg) and P10 (19.40 kg) compared to M10 (17.10 kg). Feed form significantly influenced meat-to-bone ratio, with M5P5 and P10 groups exhibiting superior ratios relative to the mash-fed group. Meat quality analysis indicated significant variations (p < 0.05) in WHC and cooking loss; the M5P5 group demonstrated enhanced WHC (63.2%) and reduced cooking loss (18.4%) compared to other treatments. Proximate composition, cholesterol content, pH, and shear force were unaffected by feed form (p > 0.05).

**Conclusion:**

The blended mash-pellet diet (M5P5) effectively enhanced specific meat quality parameters, notably WHC and cooking loss, without compromising growth performance or nutrient utilization efficiency. These findings indicate potential for the strategic use of blended feeds in fat-tailed sheep production to optimize meat quality attributes, although further studies examining long-term economic and metabolic impacts are recommended.

## INTRODUCTION

Nutrition is fundamental to animal feed and dir-ectly affects the health and well-being of livestock. Providing balanced nutrition through appropriate feeding and diet regimens is crucial for optimizing both the quality and quantity of animal-derived products [1]. A proper blend of proteins, carbohydrates, fats, minerals, and vitamins in an animal’s diet is essential for growth, sustaining physiological functions, and regulating metabolic processes. Brzozowska and Oprządek [2] emphasized that efficiently utilized feed contributes to the storage of energy in the form of body fat and milk fat, which are vital for producing high-quality animal products while also supporting the overall health and productivity of ruminants. Conversely, an unbalanced diet can result in several issues, including decreased production yields, impaired reproductive performance, weakened immune responses, and incr-eased susceptibility to diseases [3]. However, these nutritional benefits are ineffective if the small intestine is compromised. The type of feed plays a crucial role in the performance and productivity of various animal species, including poultry [4], small ruminants [5], and large ruminants [6]. Studies by Calet [4] and Chewning *et al*. [7] have demonstrated that feeding cattle a pelleted diet can increase feed intake by approximately 11%, improving growth performance, but it may reduce dry matter digestibility due to changes in particle size and rumen fermentation dynamics. In addition, pelleting enhances feed efficiency (FE) by reducing waste and increasing nutrient density, although its effects on nutrient digestibility can vary depending on the feed composition and processing conditions. Bonfante *et al*. [8] emphasized that pelleted diets are particularly effective in intensive feeding systems, supporting higher weight gain and carcass yield in cattle, making them a feasible strategy for improving productivity. Furthermore, pelleting can boost nutrient digestibility and gizzard functionality but may reduce the digestibility of certain feed components. Conversely, mash feed improves palatability in sheep, positively influencing their performance and health status [8]. The choice between pelleted and mash diets can substantially affect growth rates, feed intake, carcass characteristics, and overall productivity across species.

Despite the documented advantages of pelleted diets in poultry and the benefits of mash feed for ruminants, comparative studies on the effects of feed forms on animal performance, carcass characteristics, and meat quality in sheep remain scarce. This gap highlights the need for further research to optimize feeding strategies for small ruminants while addressing their unique physiological and nutritional requirements.

Soybean groats are a valuable local protein source derived from whole soybeans. They contain over 35% crude protein (CP), making them essential for supporting growth and tissue repair in sheep [9, 10]. Their bioavailability within the small intestine is crucial for maximizing nutritional benefits. However, once ingested, soybean groats are subject to degradation by rumen microbes under acidic conditions, which can significantly reduce their biological availability. To address this challenge, protective technologies, such as formaldehyde treatment, have been developed to protect the protein content of soybean groats from rumen microbial degradation [10, 11]. Although not a recent technology, the application of 37% formaldehyde introduces a cross-linking mechanism between aldehyde molecules and soybean proteins, forming stable bonds that resist enzymatic hydrolysis in the neutral pH environment of the rumen (pH 6–7). These bonds become labile under the acidic conditions of the abomasum (pH 2–3), ensuring protein release for absorption in the small intestine. This dual functionality not only prevents excessive protein degradation in the rumen and enhances nutrient utilization and digestibility in sheep. Bonfante *et al*. [9] demonstrated that rumen-protected amino acid formulations enhance post-ruminal absorption by preventing microbial degrad-ation, thereby optimizing nutrient supplementation efficiency. This approach ensures that critical amino acids bypass rumen fermentation, maximizing their bioavailability for metabolic processes. Similarly, rumen-protected γ-aminobutyric acid supplementation does not com-promise feed palatability but improves carcass yield, endogenous antioxidant capacity, and meat quality parameters, such as water-holding capacity (WHC) and lipid stability [1].

Although previous studies have documented the benefits of pelleted and mash feeds individually in various livestock species, comparative evaluations examining the influence of different feed forms – particularly blended mash-pellet combinations – on animal performance, nutrient digestibility, and meat quality in fat-tailed sheep remain limited. This scarcity in data restricts informed decision-making regarding optimal feeding strategies aimed at enhancing both productivity and product quality in fat-tailed sheep production systems.

This study aimed to address this knowledge gap by systematically comparing the effects of three dietary feed forms – 100% mash, 100% pellet, and a 50:50 blend of mash and pellet – on nutrient digestibility, growth performance, carcass characteristics, and meat quality attributes in fat-tailed sheep. The outcomes of this research seek to inform effective nutritional management strategies, potentially contributing to improved economic returns and meat quality standards within sheep production systems.

## MATERIALS AND METHODS

### Ethical approval

This study was approved by the Research Ethics Committee of the Universitas Sebelas Maret (Approval No. 253), ensuring compliance with ethical standards.

### Study period and location

The study was conducted from April to November 2021 at Jatikuwung Experimental Feedlot Farm in Central Java, Indonesia.

### Housing and dietary treatment

This study used 15 fat-tailed sheep with an average initial body weight (IBW) of 30.25 ± 2.55 kg. The animals had an average body condition score of 3 on a 5-point scale. They were randomly allocated into individual pens measuring 1.25 × 0.50 × 1 m, with five replicates per treatment group. The dietary treatment included three feed types: 100% mash-formed feed (M10), a 50:50 mash and pelleted mixture (M5P5), and 100% pelleted feed (P10). All diets were formulated to meet the nutritional requirements of fat-tailed sheep and contained 16.50% CP, 7.07% crude fat (CF), 20.67% crude fiber (CFi), 18.03% ash, 37.72% nitrogen-free extract (NFE), 88.07% dry matter, 81.97% organic matter, and 66.8% total digestible nutrients. The feeding trial lasted for 90 days, with diets tailored to the animal’s body weights in accordance with published standard guidelines [12]. The feeding formula used is detailed in Table 1.

**Table 1:** Nutritional content of the ingredients.

Feed ingredients	Dry matter	% Dry matter						

		CFi	CP	CF	Ash	NEFF	OM	TDN
Fermented rice straw	88.95	30.39	10.81	0.83	24.07	33.90	75.93	51.45
Protected soybean groats	86.41	7.01	36.39	11.65	7.51	37.44	92.49	88.68
Nutrifeed	88.83	24.09	12.06	7.00	20.70	36.15	79.30	62.81
Premix	95.36	-	-	-	92.65	-	-	-
Molases	77.00	7.7	4.2	0.2	0.20	87.70	99.80	76.82
Salt/Sodium chloride	95.44	-	-	-	92.64	-	-	-

**Feed form**		**Treatments^1^**						

		**M10**		**M5P5**		**P10**		

Complete feed in mash		100		50		0		
Complete feed in pallets		0		50		100		

CFi=Crude fiber, CP=Crude protein, CF=Crude fat, NEFF=Net energy for fat and fiber, OM=Organic matter, TDN=Total digestible nutrients.

1 M10=Complete feed for fat-tailed sheep that is made into 100% mash formed, M5P5=Complete feed for fat-tailed sheep that is made into a 50:50 mixture of mash and pelleted formed, P10=Complete feed for fat-tailed sheep that is made into 100% pelleted feed

The formulation of rumen-protected soybean groats begins with sun-drying to achieve an optimal moisture content. Once adequately dried, the groats are ground into fine flour. A 37% formaldehyde solution is then prepared by mixing formaldehyde with water at a 1:10 ratio. This solution is introduced into a mixing machine and sprayed onto soybean flour to ensure uniform coverage. After the application of formaldehyde, the treated soybean groats were allowed to mature for 24 h to facilitate cross-linking. After maturation, the groats are aerated for an additional 24 h to remove excess formaldehyde. In the subsequent feed manufacturing process, ingredients are weighed according to specified proportions and mixed in a mixer until a homogeneous blend is achieved. The mixture is then divided into two portions: One portion is processed through a pellet mill, and the other is used as mash feed. All feed products were subsequently sun-dried until they reached a constant weight before being packaged into sacks for storage.

### Feed consumption and nutrient digestibility

Feed consumption was evaluated using several parameters, including organic matter intake (OMI), dry matter intake (DMI), CP, CF, CFi, and non-NFE (NNFE), following the protocol established by. The daily feed intake was determined by measuring the daily feed provided to each sheep and subtracting the weight of the remaining feed after 24 h. Each sheep was individually housed with unrestricted access to feed and water. To ensure accuracy, any uneaten feed was collected, dried in a forced-air oven at 60°C for 24 h to remove moisture, and then weighed. DMI was calculated as the difference between the total feed offered and the dry weight of the leftover feed, allowing for precise analysis of feeding behavior and nutrient use throughout the study.

The OMI of the feed samples was assessed through a combustion process, where the dried samples were incinerated in a muffle furnace at 550°C for 4 h. The residual ash was weighed, and the OMI was calculated by subtracting the ash weight from the initial dry weight, adhering to protocols established by the Association of Official Analytical Chemists (AOAC) for evaluating digestibility and nutritional value. The CP content was measured using the Kjeldahl method, which involves digesting approximately 0.5 g of dried feed with concentrated sulfuric acid and a catalyst to convert organic nitrogen into ammonium sulfate. The resulting solution was distilled with sodium hydroxide to release ammonia, which was quantified through titration with standard sodium hydroxide solution; CP content was derived by multiplying total nitrogen by 6.25.

The CF content was analyzed using ether extraction, where approximately 2 g of dried sample underwent continuous extraction with petroleum ether in a Soxhlet apparatus for 6 h. The solvent from the fat extract was evaporated using a rotary evaporator, and the residue was dried at 60°C until a constant weight was achieved. CF percentage was calculated based on weight differences before and after extraction, providing an accurate measure of lipid content. CFi content was determined using a modified method in which approximately 1 g of dried sample underwent sequential treatment with dilute sulfuric acid and sodium hydroxide to eliminate soluble carbohydrates and proteins. The remaining residue was dried at 105°C until constant weight and ashed at 550°C for 4 h to assess the insoluble ash content. The CFi percentage was calculated as the difference between the initial weight and the total weight of the ash and soluble materials removed during processing. The NNFE was computed using a formula derived from the proximate analysis results. The digestibility coefficients (DCs) for nutrients such as DMI, OMI, CP, CF, CFi, and NNFE were calculated using the following formula:

DC = [Consumed Nutrient Fecal Excretion/Consumed Nutrient] × 100

Nutrient consumption is defined as the quantity of nutrients supplied minus the amount left uneaten. Total weight gain (TWG) was calculated by comparing the IBW recorded on Day 1 with the final body weight measured on day 90. Average daily gain (ADG) was calculated by dividing the TWG by the 90-day feeding period. The weights of fat-tailed sheep were recorded in the mornings following a 16-h fasting period to ensure accuracy. The FE was assessed by examining the ratio of ADG to DMI.

### Animal performance

The FE Ratio (FER) was calculated as FER = (ADG, kg)/Average Daily Feed Intake (kg), providing daily insights into the sheep’s ability to convert feed into body mass. This metric complements the traditional feed conversion ratio by emphasizing temporal efficiency. In addition, Feed Cost per Gain (FCG) was determined using FCG = Total Feed Cost (IDR)/Total Body Weight Gain (kg), evaluating the economic viability of each feeding strategy. These calculations enabled the simultaneous assessment of biological efficiency and cost-effectiveness, which are critical for optimizing livestock production systems.

### Carcass traits and sampling

After a 90-day finishing period, the animals were euthanized to assess the effects of the dietary treatments on carcass characteristics. Evaluations included hot carcass weight, backfat thickness, and rib eye area to determine carcass weight and grade. Carcass quality was assessed according to Indonesian-National Standards based on marbling score, meat and fat color, firmness, and maturity. Four muscles were excised from each animal 24 h post-mortem, vacuum-sealed, and transported to the laboratory. Samples were stored overnight at 2°C ± 2°C. Each muscle was then cut into six 2-cm-thick steaks. Three steaks from each animal were placed on Styrofoam trays, overlapped with oxygen-permeable film, and stored in darkness at 5°C ± 0.5°C for 3 and 6 days to evaluate the influence of storage on color, pH, and lipid oxidation. The remaining steaks were reserved for cooking loss and shear force analyses, as well as color, pH, and WHC assessments.

### Proximate composition

Ground samples were processed using a food grinder (model HMF-1600 PB, Hanil Electric, Seoul, South Korea) at moderate speed for 10 s. Proximate composition analysis was conducted following standardized AOAC methods. Moisture content was determined by oven-drying samples at 105°C for 24 h. CF was analyzed using ether extraction in a Soxhlet apparatus (Pyrex, Corelle Brands LLC, Illinois, USA). Nitrogen content was measured using a Kjeltec system (2200 Kjeltec Auto Distillation Unit, Foss, Hilleroed, Sweden), and CP was calculated by multiplying nitrogen content by 6.25. Crude ash was quantified by incinerating samples in a muffle furnace at 550°C for 12 h. These methods ensured accurate and reliable determination of the nutritional composition of the samples, adhering to internationally recognized protocols for food and feed analysis.

### Cholesterol content

The cholesterol content in this study was determined using the method described by Barido *et al*. [10]. A 2-g sample was spiked with 5-cholestane as an internal standard and was saponified with 95% ethanol and 50% potassium hydroxide (KOH) at 80°C for 70 min under reflux. After cooling, the hydrolyzed mixture was extracted with n-hexane and washed sequentially with KOH solutions and water. The purified extract, dissolved in dimethylformamide, was analyzed by gas chromatography using an Agilent 7890 N system with an HP-5 column (30 m × 0.33 mm × 0.25 mm). Helium was used as the carrier gas (1.0 mL/min, 1:12.5 split ratio). The injector and flame ionization detector temperatures were 250°C and 300°C, respectively. The column temperature program included an initial hold at 190°C, followed by ramps to 230°C and 270°C, with a final hold at 270°C. Cholesterol was quantified by comparing the target peak area with the internal standard (mg/100 g meat).

### WHC, cooking loss, and shear force

The WHC of meat was assessed using a modified centrifugation method in which ground samples were heated at 75°C for 30 min, cooled for 30 min, and centrifuged at 980× *g* for 10 min at 24°C. This approach aligns with established protocols that utilize centrifugal force to measure water exudate, ensuring an accurate evaluation of WHC [13]. Cooking loss was calculated as the percentage difference between the initial weight (W1) and post-cooking weight (W2), reflecting moisture retention during cooking. The Warner-Bratzler shear force test was used to assess meat tenderness, using standardized parameters for cutting speed and sample dimensions to ensure precision and repeatability.

### Statistical analysis

Statistical analyses were conducted using R software (R Foundation, version 3.6.1, Vienna, Austria). Data normality and homogeneity of variances were initially verified using the Shapiro–Wilk test and Levene’s test, respectively. Subsequently, a one-way analysis of variance was performed to determine the effects of different feed form treatments (M10, M5P5, and P10) on nutrient digestibility, animal performance parameters, carcass characteristics, and meat quality attributes. When significant effects were detected, treatment means were compared using Tukey’s Honestly Significant Difference *post hoc* test. Results were considered statistically significant at a probability level of p < 0.05. All data are expressed as means ± standard error of the mean.

## RESULTS

### Feed consumption

Table 2 summarizes the influence of feed form on nutrient intake in fat-tailed lamb. Although numerical variations were observed across feed forms, no statistically significant differences (p > 0.05) were detected for any intake parameters. The DMI, OMI, CP, CF, CFi, and NEFF did not differ among treatments (p > 0.05). The net energy for fat and fiber (NEFF) across the various feed types studied had no significant differences among feed forms.

**Table 2:** Effects of feed form on the consumption of fat-tailed lambs.

Consumption (g/head/day)	Treatments^1^			p-value	Significance

	M10	M5P5	P10		
DMI	1415.21 ± 215.75	1694.24 ± 194.39	1571.18 ± 235.24	0.16	NS
OMI	1155.09 ± 176.10	1382.84 ± 158.66	1282.4 ± 192.00	0.12	NS
CP	233.46 ± 35.59	279.49 ± 32.07	259.19 ± 38.81	0.22	NS
CF	100.11 ± 15.26	119.84 ± 13.75	111.14 ± 16.64	0.43	NS
CFi	292.61 ± 44.61	350.31 ± 40.19	324.86 ± 48.64	0.18	NS
NEFF	528.91 ± 80.63	633.19 ± 72.65	587.20 ± 87.92	0.16	NS

DMI=Dry matter intake, CFi=Crude fiber, CP=Crude protein, CF=Crude fat, NEFF=Net energy for fat and fiber, OMI=Organic matter intake, NS=Not significant.

1 M10=Complete feed for fat-tailed sheep that is made into 100% mash formed, M5P5=Complete feed for fat-tailed sheep that is made into a 50:50 mixture of mash and pelleted formed, P10=Complete feed for fat-tailed sheep that is made into 100% pelleted feed

### Nutrient digestibility

This study evaluated the effects of different feed forms on nutrient intake in fat-tailed sheep (Table 3), revealing no significant differences (p > 0.05). These results suggest consistent nutrient consumption across feed forms, with CFi intake levels supporting rumen function and microbial fermentation. However, the CFi differed significantly (p < 0.05). This variation highlights the potential for optimizing energy intake through tailored feed formulations. While most metrics remained unaffected by feed form, the CFi results underscore the importance of strategic dietary design to enhance nutrient digestibility and growth performance in fat-tailed sheep.

**Table 3:** Effects of feed form on nutrient digestibility in fat-tailed lamb.

Consumption (g/head/day)	Treatments^1^			p-value	Significance

	M10	M5P5	P10		
DMI	43.08 ± 1.76	44.98 ± 0.94	43.75 ± 2.33	0.27	NS
OMI	43.31 ± 1.96	45.56 ± 1.26	44.47 ± 2.40	0.23	NS
CP	71.21 ± 8.26	77.89 ± 6.45	74.10 ± 3.86	0.30	NS
CF	83.97 ± 5.91	87.19 ± 2.44	86.49 ± 1.69	0.40	NS
CFi	59.27 ± 2.51^b^	65.11 ± 3.87^a^	64.11 ± 1.21^ab^	0.01	S
NEFF	14.48 ± 2.62	12.60 ± 3.11	12.59 ± 2.96	0.40	NS

DMI=Dry matter intake, CFi=Crude fiber, CP=Crude protein, CF=Crude fat, NEFF=Net energy for fat and fiber, OMI=Organic matter intake, NS=Not significant, S=Significant.

1 M10=Complete feed for fat-tailed sheep that is made into 100% mash formed, M5P5=Complete feed for fat-tailed sheep that is made into a 50:50 mixture of mash and pelleted formed, P10=Complete feed for fat-tailed sheep that is made into 100% pelleted feed.

^a,b^Means within the same row indicating statistical significance among feed forms (p < 0.05)

### Animal performance

The animal performances following feeding treatments with different forms of feed in fat-tailed sheep are illustrated in Table 4. The evaluated parameters included initial and final weights, weight gain, ADG, FER, and feeding cost per gain. Initial weights were similar across treatments, establishing a consistent baseline for growth assessment. After a 90-day feeding trial, there were no significant differences (p > 0.05) in final weights. Similarly, the ADG did not significantly vary among the groups (p > 0.05). The FER was also comparable across treatments, indicating no superior feed conversion efficiency for any feed type. Feeding cost per gain ranged from Rp 44,635 to Rp 46,651 (United States Dollar 2.71–2.84) across treatments.

**Table 4:** Effects of feed form on the production performance of fat-tailed lamb.

Consumption (g/head/day)	Treatments^1^			p-value	Significance

	M10	M5P5	P10		
Initial weight (kg)	22.42 ± 3.86	24.38 ± 2.91	23.25 ± 2.23	0.67	NS
Final weight (kg)	35.80 ± 4.00	40.12 ± 3.65	37.92 ± 1.21	0.25	NS
Weight gain (kg)	13.38	15.74	14.67	0.95	NS
Average daily gain (g/day)	136.53 ± 34.22	160.61 ± 36.66	149.69 ± 33.80	0.41	NS
FER (%)	10.94 ± 3.47	10.23 ± 1.89	10.45 ± 2.56	0.13	NS
Feed cost per gain (Rp)	46.651	44.635	46.561	0.21	NS

FER=Feed efficiency ratio, NS=Not significant.

1 M10, complete feed for fat-tailed sheep that is made into 100% mash formed, M5P5=Complete feed for fat-tailed sheep that is made into a 50:50 mixture of mash and pelleted formed, P10=Complete feed for fat-tailed sheep that is made into 100% pelleted feed

### Carcass characteristics

Table 5 highlights the significant impact of feed form on carcass characteristics and meat quality in fat-tailed sheep. Hot carcass weight varied significantly among the feed forms(p < 0.05). The lower hot carcass weight in the M10 group underscores the superior efficiency of pelleted or mixed feed forms in enhancing meat yield, which is consistent with findings that dietary strategies can optimize growth performance and production efficiency [1, 4]. In contrast, backfat thickness and rib eye area showed no statistically significant differences among treatments (p > 0.05). These results suggest that feed form had a minimal influence on muscle deposition and fat distribution, consistent with studies indicating that carcass traits may not always correlate directly with feeding regimens. The meat-to-bone ratio, however, demonstrated significant variation, indicating that feed form can influence carcass composition, potentially improving the marketability of meat products. Meanwhile, the fleshing index showed no significant differences across groups, further supporting the notion that all feeding strategies were effective in maintaining overall animal condition and market readiness.

**Table 5:** Carcass characteristics of fat-tailed lamb according to feed form.

Consumption (g/head/day)	Treatments			p-value	Significance

	M10	M5P5	P10		
Yield traits					
Hot carcass weight (kg)	17.10 ± 0.46^a^	19.57 ± 1.15^b^	19.4 ± 1.00^b^	0.02	S
Backfat thickness (inch)	0.15 ± 0.02	0.14 ± 0.03	0.18 ± 0.04	0.37	NS
Rib eye area (cm^2^)	22.83 ± 2.90	26.67 ± 2.47	30.92 ± 6.29	0.14	NS
Yield grade	1.89 ± 0.16	1.80 ± 0.34	2.15 ± 0.35	0.37	NS
Meat bone ratio	2.27 ± 0.10^a^	248 ± 004^b^	2.46 ± 0.06^b^	0.02	NS
Fleshing index	0.22 ± 0.03	0.25 ± 0.02	0.24 ± 0.01	0.39	NS

NS=Not significant, S=Significant. ^1^M10=Complete feed for fat-tailed sheep that is made into 100% mash formed, M5P5=Complete feed for fat-tailed sheep that is made into a 50:50 mixture of mash and pelleted formed, P10=Complete feed for fat-tailed sheep that is made into 100% pelleted feed. ^a,b^Means within the same row indicating statistical significance among feed forms (p < 0.05)

### Meat quality

#### Effects of feed form on proximate composition and cholesterol levels

The meat quality parameters of fat-tailed sheep across various muscle groups are presented in Table 6. The moisture content was assessed for Biceps femoris (BF), Pectoralis profundus (PP), Triceps brachii (TB), and Longissimus dorsi (LD) muscles. As shown in Table 6, moisture levels for M10, M5P5, and P10 treatments were as follows: BF (70.59%, 70.17%, 67.99%), PP (65.55%, 65.55%, 68.27%), TB (69.34%, 68.02%, 66.13%), and LD (66.58%, 68.04%, 68.85%), whereas statistical analysis revealed no significant differences (p > 0.05), indicating that feed form did not substantially influence moisture levels across muscle groups. The meat CP percentages also showed no significant differences (p > 0.05). Similarly, meat fat content varied across muscle types but remained statistically insignificant (p > 0.05). In addition, cholesterol levels ranged from 33.48 to 37.91 mg/dL across treatments and muscle groups, with no significant differences observed (p > 0.05). These findings suggest that feeding strategies did not adversely affect the nutritional composition of lamb meat. The pH values, ranging from 6.55 to 7.06, were consistent across treatments, further indicating the negligible impact of feed form on meat acidity (Table 7). However, the WHC was significantly influenced by feed form in the PP and LD muscles, with pelleted feed (P10) exhibiting a higher WHC compared with the M10 and M5P5 treatments (p < 0.05). No significant differences were found in WHC between the BF and TB muscles. In terms of cooking loss, it exhibited inconsistent trends, with the lowest CL percentages recorded for P10 in the BF, M5P5 in the TB, and M10 in the PP muscles, although these variations lacked a clear pattern across treatments. Texture profiles, including tenderness, did not differ among feeding regimens across all muscle samples.

**Table 6:** Proximate composition and cholesterol levels of meat derived from fat-tailed lamb according to feed form.

Parameters	Muscles	Treatments^1^			Significance

		M10	M5P5	P10	
Moisture (%)	BF	70.59	70.17	67.99	NS
	PP	65.55	65.55	68.27	NS
	TB	69.34	68.02	66.13	NS
	LD	66.58	68.04	68.85	NS
Crude protein (%)	BF	24.95	25.30	25.57	NS
	PP	25.08	23.53	23.58	NS
	TB	25.15	25.19	24.98	NS
	LD	26.10	26.68	25.48	NS
Crude fat (%)	BF	4.50	4.33	5.32	NS
	PP	6.63	6.88	6.39	NS
	TB	7.20	5.73	5.80	NS
	LD	6.11	5.69	4.55	NS
Cholesterol	BF	33.75	34.99	34.68	NS
	PP	37.91	37.72	34.89	NS
	TB	33.62	35.63	33.48	NS
	LD	35.33	35.68	37.04	NS

TB=Triceps brachii, BF=Biceps femoris, PP=Pectoralis profundus, LD=Longissimus dorsi, NS=Not significant.

1 M10, complete feed for fat-tailed sheep that is made into 100% mash formed, M5P5=Complete feed for fat-tailed sheep that is made into a 50:50 mixture of mash and pelleted formed, P10=Complete feed for fat-tailed sheep that is made into 100% pelleted feed

**Table 7:** Quality properties of meat derived from fat-tailed lamb according to feed form.

Parameters	Muscles	Treatments^1^			Significance

		M10	M5P5	P10	
pH value	BF	7.06	7.01	6.80	NS
	PP	6.55	6.55	6.83	NS
	TB	6.94	6.80	6.61	NS
	LD	6.66	6.80	6.89	NS
WHC (%)	BF	44.23	46.66	42.52	NS
	PP	32.91^b^	32.27^b^	39.10^a^	S
	TB	38.28^a^	30.54^b^	40.57^a^	S
	LD	39.54^b^	36.16^b^	39.98^a^	S
Cooking loss (%)	BF	28.17^a^	28.60^a^	20.24^b^	S
	PP	13.43^b^	17.54^a^	17.07^a^	S
	TB	17.38^a^	9.07c	15.15^b^	S
	LD	13.43^a^	22.73^a^	22.23^a^	S
Shear force value (kgf)	BF	7.59	8.09	7.31	NS
	PP	6.78	6.94	6.42	NS
	TB	6.21	7.99	8.19	NS
	LD	7.28	7.62	6.66	NS

TB=Triceps brachii, BF=Biceps femoris, PP=Pectoralis profundus, LD=Longissimus dorsi, WHC=Water-holding capacity, NS=Not significant, S=Significant.

1 M10=Complete feed for fat-tailed sheep that is made into 100% mash formed, M5P5=Complete feed for fat-tailed sheep that is made into a 50:50 mixture of mash and pelleted formed, P10=Complete feed for fat-tailed sheep that is made into 100% pelleted feed.

^a,b,c^Means within the same row indicating statistical significance among feed forms (p < 0.05)

## DISCUSSION

### Feed consumption and nutritional strategies

The present study found no significant differences in feed consumption among the different feed forms tested. Nevertheless, feed consumption remains a critical factor in ensuring nutrient availability, which directly influences growth rates and overall deve-lopment. Protein, as a limiting nutrient, plays a key role in muscle development and tissue repair by sup-plying the essential amino acids required for growth [1]. Factors such as palatability and digestibility significantly affect feed intake. Palatability determines the willingness of fat-tailed sheep to consume feed, whereas digestibility governs the effectiveness with which nutrients are absorbed and utilized [14]. Higher OMI is essential to meet the metabolic demands of growing fat-tailed sheep, supporting optimal health outcomes [1]. Fiber content also plays a crucial role in digestibility, as it promotes healthy rumen function and enhances nutrient absorption efficiency. This study observed no significant influence of feed form on fiber content, which is consistent with previous research findings by Malla *et al*. [5] and Vukmirović *et al*. [8]. Conversely, energy availability was significantly affected by feed type, with feeds high in digestible energy promoting better weight gain and overall performance by providing readily available nutrients for metabolic processes. These findings underscore the importance of optimizing feeding strategies that balance energy and nutrient availability to improve productivity and health outcomes in fat-tailed sheep.

### Nutrient digestibility and economic viability

Economic metrics are essential for the financial viability of lamb feed regimens. Minor variations in feed costs highlight the need to balance economic factors with growth performance when selecting feeding strategies [15]. This study found no significant differences in overall animal performance (weight gain, ADG). However, differences in FE and cost-effectiveness emphasize the critical role of tailored nutritional strat-egies in optimizing lamb performance and enhan-cing economic returns. This relationship can be expla-ined by the fact that more efficient feed utilization leads to better weight gain per unit of feed consumed, ultimately reducing production costs [1].

Carcass weight is a critical determinant in the meat industry because heavier carcasses typically yield more marketable meat, directly influencing producers’ profitability. The significant differences observed in hot carcass weight indicate that specific feeding strategies can be optimized to maximize growth potential in fat-tailed sheep. In examining backfat thickness, measure-ments ranged from 0.14 to 0.18 inches, with p = 0.37, indicating no significant differences among treatment groups. This finding indicates that although fat-tailed sheep may gain weight, the nutritional strategies emp-loyed did not lead to excessive fat deposition, which was consistent with the results of a previous study by De Jong *et al*. [16]. Meanwhile, maintaining an optimal backfat level is essential for ensuring that fat-tailed sheep meet market specifications while promoting healthy growth. Excessive fat deposition can reduce carcass value, whereas insufficient fat may compromise meat quality and consumer appeal. In this study, the levels of backfat remained within acceptable ranges across treatment groups, demonstrating that the feeding strategies effectively balanced growth and fat deposition.

The rib eye area, which serves as an indicator of muscle development and overall carcass quality, showed trends toward larger areas in certain feed groups, although these differences were not statistically significant (p = 0.14). This observation suggests that specific feed forms may enhance muscle growth more effectively than other forms [17]. The underlying mech-anism could involve optimization of specific amino acids or energy sources that prioritize muscle hypertrophy over fat accumulation. Since the rib eye area correlates strongly with both meat yield and consumer preference for premium cuts, further research into dietary inter-ventions that promote muscle development could enh-ance carcass value and marketability [18].

Further, yield grade, which reflects the proportion of usable meat relative to fat and bone, is another critical parameter for evaluating carcass quality. This study revealed a substantial influence of feed form on the meat-to-bone ratio, with higher ratios indicating a greater proportion of edible meat relative to bone weight. These findings are particularly important for producers because a higher meat-to-bone ratio directly enhances profitability by increasing the yield of marketable meat. These results suggest that different forms of complete feed do not negatively affect carcass characteristics in fat-tailed sheep. In addition to confirming a previous study by Gjorgjievski *et al*. [19], this study by Kashan *et al*. [20] also highlights the necessity for targeted nutritional strategies that not only promote growth but also improve feed utilization efficiency in producing high-quality lamb. Understanding how various feeding strategies affect this index is crucial for producers aiming to optimize both animal welfare and economic returns.

In terms of meat quality, maintaining optimal moisture content is critical for meat quality because it directly impacts juiciness and overall palatability, which are key factors influencing consumer acceptance and marketability of lamb products [21]. This study by Lee *et al*. [1] demonstrated stable moisture levels across muscle groups, indicating that the nutritional strategies employed effectively preserved this essential attribute. High protein levels, which are desirable for supporting lean muscle mass during the rapid post-weaning growth phase, were also observed. Protein plays a key role in muscle repair and development, which is consistent with the consumer demand for high-quality, nutrient-dense meat [22]. The stability of fat content across different dietary formulations (mash vs. pellet) highlights the flexibility in feed selection without compromising energy density or flavor profiles – both critical to consumer preferences [23]. Cholesterol content, an important consideration for health-conscious consumers due to its association with cardiovascular health, also showed no significant variation across feeding regimens. These findings suggest that producers can adopt diverse nutritional strategies while maintaining essential meat quality attributes, such as moisture, CP, CF, and chol-esterol levels [24]. The pH values across muscle groups (BF, PP, TB, and LD) remained stable regardless of the feeding regimen. This stability is crucial for maintaining post-slaughter meat quality by influencing glycolysis rates and lactic acid production, which affect tenderness, shelf life, and sensory attributes such as color and flavor. Elevated pH levels can lead to undesirable qualities such as pale, soft, and exudative meat [25].

The WHC is another critical parameter affecting juiciness and tenderness, and it exhibited significant variation among muscle groups. For example, BF showed the highest WHC. The WHC is influenced by factors such as protein structure, fat content, and connective tissue presence. Higher WHC is correlated with improved sensory qualities by enhancing juiciness and reducing cooking losses, attributes highly valued by consumers [8]. The observed differences in WHC suggest that specific feeding regimens may promote better moisture retention in certain muscles, providing opportunities to optimize meat quality further. In the present study, the cooking loss percentage varied significantly among the muscle groups. Lower cooking losses indicate better moisture retention and higher yields from cuts, enhancing both sensory attributes and economic value. Consumers typically prefer products that maintain weight after cooking due to their superior texture and flavor. These findings suggest that feeding strategies can influence moisture retention during cooking processes, which is a crucial consideration for producers aiming to enhance product quality while maximizing profitability [23]. Finally, shear force, a widely recognized measure of tenderness, showed no significant differences across feeding regimens or muscle groups. Tenderness remains a critical quality attribute that influences consumer preferences; tougher meats are less desirable in culinary applications. Factors such as muscle fiber composition, slaughter age, and post-mortem handling practices play significant roles in determining tenderness. Although this study by Nati-onal Research Council *et al*. [12] found no significant variation in shear force values across treatments, further research into specific dietary components could uncover potential interventions to improve tenderness through nutritional strategies.

## CONCLUSION

The present study systematically evaluated the effects of dietary feed forms (pelleted, mash, and a 50:50 blended mash-pellet) on nutrient digestibility, animal performance, carcass characteristics, and meat quality in fat-tailed sheep. Results demonstrated that feed form did not significantly affect feed intake, nutrient digestibility, or animal performance metrics such as ADG and FER. However, notable differences were observed in carcass traits, with blended mash-pellet (M5P5) and fully pelleted (P10) diets yielding significantly greater hot carcass weights and superior meat-to-bone ratios compared to the mash-only diet (M10). Furthermore, meat quality parameters, including WHC and cooking loss, were significantly improved in the blended diet group, indicating enhanced meat juiciness and tenderness.

A key strength of this study lies in its systematic comparison of distinct feed forms under controlled experimental conditions, providing robust evidence for the impacts of feed form on carcass yield and meat quality in fat-tailed sheep. This comparative approach fills a critical knowledge gap and offers practical insi-ghts into optimizing sheep production strategies for enhanced meat quality outcomes.

Nonetheless, several limitations must be acknowledged. First, the relatively small sample size of animals per treatment group might have restricted the statistical power to detect subtle differences among treatments. In addition, economic implications, including comprehensive cost-benefit analyses of the feed forms, were not exhaustively examined, limiting the extrapolation of findings to broader production contexts.

Future research should focus on expanding sample sizes to strengthen statistical validity and conducting comprehensive economic evaluations of feed form strategies. Long-term studies investigating metabolic impacts, animal welfare considerations, and consumer sensory preferences related to different feed forms are recommended. Furthermore, exploring the underlying biological mechanisms contributing to improved meat quality attributes, particularly WHC and cooking loss, could provide additional insights to optimize feeding practices and enhance product marketability.

## AUTHORS’ CONTRIBUTIONS

JR, AP, SDW, SS, MC, and FHB: Conceptualization, methodology, writing – review and editing, visualization, and supervision. JR, WNY, AAA, and GRJP: Software, formal analysis, investigation, and data curation. JR, AP, SDW, SS, MC, WNY, AAA, GRJP, and FHB: Validation. JR: Resources, project administration, and funding acquisition. JR and FHB: Writing–original draft preparation. All authors have read and approved the final manuscript.
